# Optimization of Fermentation Conditions to Enhance Cytotoxic Metabolites Production by *Bacillus velezensis* Strain RP137 from the Persian Gulf

**Published:** 2020

**Authors:** Roya Pournejati, Hamid Reza Karbalaei-Heidari

**Affiliations:** Molecular Biotechnology Laboratory, Department of Biology, Faculty of Science, Shiraz University, Shiraz, Iran

**Keywords:** A549 cells, Bacillus, Industrial development, Liver neoplasms

## Abstract

**Background:**

Isolation, introduction and producing bioactive compounds from bacteria, especially marine bacteria, is an attractive research area. One of the main challenges of using these metabolites as drug and their industrialization is the optimization of production conditions.

**Methods:**

In the present study, the response surface methodology was applied to optimize the production of a cytotoxic extract (C-137-R) by *Bacillus velezensis* (*B. velezensis*) strain RP137. Initially, among the three carbon and three nitrogen sources, rice starch and potassium nitrate were selected as the best, with cell toxicity equal to IC50=54.4 and 45.1 *μg/ml* in human lung and liver cancer cell lines, respectively (A549 and HepG2). In the next step, fractional factorial design was performed to survey effect of seven physical and chemical factors on the amount of production, and the most important factors including carbon and nitrogen sources with the positive effect and the sea salt with negative effect were determined. Finally, using the central composite design with 20 experiments, the best concentrations of rice starch and potassium nitrate (1.5%) and sea salt (1%) were obtained.

**Results:**

The average amount of dried extract produced in the optimum conditions was 131.1 *mg/L* and the best response was 71.45%, which is more than 28-fold better than the pre-optimized conditions.

**Conclusion:**

In general, it can be suggested that the use of modern statistical methods to optimize environmental conditions affecting the growth and metabolism of bacteria can be a highly valuable tool in industrializing the production of bioactive compounds.

## Introduction

The secondary or “specialized” metabolites produced by microorganisms have helped human being to overcome many diseases and have been a pillar of modern medicine since 21^st^ century. Secondary metabolites are molecules which are not necessary for growth of an organism, at least under laboratory conditions; however, they reveal significant survival advantages in nature. For instance, they play a role as defense or signaling molecules in ecological interactions 1, as elements required for self-toxicity in a form of programmed cell death processes prior to morphological differentiation 2, and they help the microorganism to enhance chemical defense when needed. Many researchers have focused on screening and production optimization of these molecules from natural sources such as microorganisms with goals of pharmacological applications, mostly as antibiotics or antitumor compounds 3–6.

Secondary metabolism is usually activated when environmental condition changes or starvation occurs to microorganism at the end of logarithmic phase of growth, then a complex series of changes in its global gene expression happen, leading to secondary metabolite production and morphological differentiation 7. Therefore, expression of secondary metabolic gene clusters could have been manipulated by environmental and developmental changes 8,9.

Microbial fermentations are widely applied to industrialize these valuable metabolites where the appropriate conditions for secondary metabolite production and lowering the cost are highly desirable. Secondary metabolites originated from intermediates or precursors which are produced by primary/intermediate metabolism 10 are branching points of biosynthetic pathways leading to synthesize an end product. Accordingly, effect and interaction between nutrients is a major determinant of secondary metabolism fluctuations. Moreover, secondary metabolism is regulated by precursors, carbon sources, nitrogen sources, phosphate, trace elements, *etc* 11,12.

Regulation of secondary metabolism can be achieved at different levels, starting with the regulation of extracellular nutrients and minor alterations in the composition of fermentation media 13,14. Designing an appropriate medium and optimizing the conditions for microorganism cultivation are of great importance for enhancing secondary metabolite production 15. To do so, several statistical approaches such as Response Surface Methodology (RSM) are widely utilized. Response surface methodology is a suitable mathematical and statistical tool and is extensively used for optimization of microbial compounds production by many researchers 16–20. The use of RSM to optimize fermentation conditions is mostly dependent on the following experimental steps 21. First, they include using the fractional factorial design to distinguish significant factors of the fermentation process and then, performing the Central Composite Design (CCD) to establish fermentation model and optimal fermentation conditions.

In our previous study, an aminoglycoside class of antibiotic named S-137-R was introduced from aqueous fraction of the initial methanolic extract of *Bacillus velezensis (B. velezensis)* strain RP137 22. The ethyl acetate fraction (C-137-R) of the original methanolic extract showed a cytotoxic property against cancerous cells as well. So, in the present study, an attempt was made to optimize production of this cytotoxic fraction and investigate the combined influence of multiple factors of cultivation conditions including chemical (nutrient compositions) and physical (cultivation conditions) parameters using the fractional factorial design and response surface methodology under Design Expert 8.0.7 software platform.

## Materials and Methods

### Materials

Rice starch was purchased from KeshtSabz, Shiraz, Iran. Peptone and yeast extract were obtained from Difco, Themofisher scientific. Potassium nitrate and ammonium sulfate were purchased from Merck. A549, HepG2, MCF-7 and Hela cells were obtained from Pasteur Institute, Tehran, Iran. The SV-80 cell as a normal human fibroblast cell line was purchased from Cell Lines Service (CLS) GmbH, Eppelheim, Germany. All the solvents were purchased from Samchun Pure Chemical Co., Seoul, Republic of Korea with appropriate grade and used with no further purification.

### Bacterial culture and organic extraction

*B. velezensis* strain RP137 was isolated from the Persian Gulf shoreline, Hormozgan province, Iran. The strain RP137 was identified based on morphological, biochemical and 16S ribosomal RNA gene sequence and deposited to Persian Type Culture Collection (PTCC 1927). The isolated RP137 cells were primarily cultured in Tryptic soy broth (TSB, Merck) as basal medium and then inoculated into the production media consisting of various carbon and nitrogen sources. After cultivation of the strain RP137 at 30°*C* and 180 *rpm* for 7 days, the biomass was collected by centrifugation and treated with acidic methanol (1:24 V/V, HCl 1M: absolute methanol) for 45 *min*, 3 times. After evaporation of the methanol, collected cell extracts were dissolved in ethyl acetate and the cytotoxic effect of soluble fractions was tested against A459 (Human lung adenocarcinoma) cells as described later.

### Optimization procedures and experimental design Carbon and nitrogen source selection

Six different media containing 10 *g/l* of each of three low-cost carbon sources (wheat flour, rice starch and molasses) and three nitrogen sources (5 *g/l* (NH_4_)_2_ SO_4_, 10 *g/l* KNO_3,_ and peptone) were designed and supplemented with 1 *g/l* of yeast extract as vitamin source. Inoculation was done by transferring 300 *μl* of 3 day seed culture to 30 *ml* of the above mentioned media in 100 *ml* Erlenmeyer flasks. Cultivations were conducted at 30*°C*, and 180 *rpm* for 7 days. All experiments were performed at least in triplicate.

### Fractional factorial design

For identification of the most significant variables affecting the cytotoxic compound production, six variables (X1/rice starch, X2/KNO_3_, X3/sea salt, and X4/pH, X5/inoculation size and X6/incubation time) were selected and analyzed by the fractional factorial (2^6−2^) design experiment. The principal effects of each variable on the response (C-137-R production) were represented at the high and the low levels. The experimental design with the variables, symbol codes, and experimental levels of the variables are shown in tables 1 and 2, respectively. The bioactive compound production value (Response) was defined by the following equation:

$$\begin{array}{l} \text{Response}\;\%=\text{cell}\;\text{death}\;(\%)\times \text{relative}\;\text{organic}\;\text{extract}\;\text{production}\\ \text{Relative}\;\text{organic}\;\text{extract}\;\text{production}=\frac{\text{organic}\;\text{extract}\;(\mathrm{mg}/\mathrm{ml})\;\text{in}\;\text{a}\;\text{culture}}{\text{highest}\;\text{amount}\;\text{of}\;\text{organic}\;\text{extract}\;(\mathrm{mg}/\mathrm{ml})\;\text{in}\;\text{experiment}} \end{array}$$

**Table 1.** Carbon and nitrogen sources selection experiments based on cytotoxic effect of the extracts on A549 cell viability. The data are the average of at least three experiments ± standard deviation

**Medium number**	**Carbon source**	**Nitrogen source**	**Biomass D.W. (*mg/l*)**	**Extract D.W.^a^ (*mg/l*)**	**IC_50_ on A549 cells (*μg/ml*)**	**IC_50_ on HepG2 cells (*μg/ml*)**
**1**	Rice starch	Ammonium sulfate	80.5±1.8	54.7±3.4	187.0±30.7	150.2±15.8
**2**	Rice starch	Potassium nitrate	127.5±11.1	86.0±8.7	54.4±10.04	45.1±12.6
**3**	Rice starch	Peptone	122±10.1	28.2±3.5	310.7±12.3	280±7.8
**4**	Wheat flour	Ammonium sulfate	133.7±9.7	48.2±3.6	57.18±11.8	50.8±12.1
**5**	Wheat flour	Potassium nitrate	207.8±5.5	31.5±8.5	93.13±22.3	87.4±9.1
**6**	Wheat flour	Peptone	85.3±3.5	59.2±12.8	132.9±19.86	120.2±6.8
**7**	Molasses	Ammonium sulfate	81.2±12.8	37.0±7.3	344.4±20.5	330±14.2
**8**	Molasses	Potassium nitrate	117.6±9.6	36.1±6.5	214.4±18.15	203.8±11.2
**9**	Molasses	Peptone	161.3±11.6	111.8±12.4	171.6±8.9	167.2±18.2

a D.W.: Dry weight.

**Table 2.** Fractional factorial experimental design and the responses. The data are the average of at least three experiments ± standard deviation

**Run**	**Starch (%)**	**KNO3 (%)**	**Sea salt (%)**	**pH**	**Inoculation (%)**	**Incubation time (days)**	**Extract D.W. (*mg/l*)**	**Cell Death HepG2 (%)**	**Cell Death A549 (%)**	**Response A549 (%)**
**1**	0.1	0.1	1	6	1	3	66.6±3.3	15.2±3.7	8.1±3.4	3.3
**2**	1	0.1	1	6	5	3	75.0±5.2	19.6±3.9	14.6±15.3	10.9
**3**	0.1	1	1	6	5	7	31.7±1.7	21.11±0 5	18.0±12.2	18.0
**4**	1	1	1	6	1	7	56.7±3.3	75.03±1.3	67.3±6.7	63.7
**5**	0.1	0.1	5	6	5	7	43.4±6.7	9.8±0.5	0	0
**6**	1	0.1	5	6	1	7	48.3±5.7	8.4±2.2	0	0
**7**	0.1	1	5	6	1	3	58.4±5.0	22.8±8.3	18.9±18.2	2.5
**8**	1	1	5	6	5	3	135.0±1.7	5.9±0.4	1.8±6.8	3. 7
**9**	0.1	0.1	1	8	1	7	30.0±0.4	3.5±1.6	0	0
**10**	1	0.1	1	8	5	7	61.7±1.7	69.3±2.6	61.1±1.0	49.6
**11**	0.1	1	1	8	5	3	73.2±16.6	54.3±2.0	48.1±0.3	49.3
**12**	1	1	1	8	1	3	41.6±11.7	3.8±3.7	0	0
**13**	0.1	0.1	5	8	5	3	51.4±5.0	8.7±10.9	0	0
**14**	1	0.1	5	8	1	3	70.0±3.4	5.9±0.38	0	0
**15**	0.1	1	5	8	1	7	55.0±11.6	6.7±0.5	5.5±6.5	4.5
**16**	1	1	5	8	5	7	176.7±6.6	0	0	0

D.W.: Dry weight.

### Response surface methodology design

Response Surface Methodology (RSM) was applied to identify optimum levels of each effective variable including X_1_ (Rice starch), X_2_ (KNO_3_) and X_3_ (Sea salt) to obtain maximum response. The coded independent variables used in the RSM design are listed in table 3. The experiments were designed according to the CCD using 20 experiments with six central points, as shown in table 4. Data were analyzed by Design Expert 8.0.7 software.

**Table 3.** Analysis of variance, fractional factorial experiments

**Source**	**Sum of squares**	**df**	**Mean square**	**Fvalue**	**p-value**
**Model**	6706.57	14	479.04	6.606E+005	0.0010
**A-carbon**	187.83	1	187.83	2.590E+005	0.0013
**B-nitrogen**	426.10	1	426.10	5.876E+005	0.0008
**C-salt**	2013.75	1	2013.75	2.777E+006	0.0004
**D-pH**	2.19	1	2.19	3019.27	0.0116
**E-Inoculation**	240.68	1	240.68	3.319E+005	0.0011
**F-Incubation**	311.93	1	311.93	4.301E+005	0.0010
**AB**	222.39	1	222.39	3.067E+005	0.0011
**AC**	171.72	1	171.72	2.368E+005	0.0013
**AD**	183.08	1	183.08	2.525E+005	0.0013
**AE**	168.14	1	168.14	2.319E+005	0.0013
**AF**	1155.72	1	1155.72	1.594E+006	0.0005
**BD**	262.72	1	262.72	3.623E+005	0.0011
**ABD**	1190.85	1	1190.85	1.642E+006	0.0005
**ABF**	169.47	1	169.47	2.337E+005	0.0013
**Residual**	7.252E-004	1	7.252E004		
**Cor total**	6706.57	15			

**Table 4.** Variables in CCD experiments in terms of coded factors and respective levels

**Coded factor**	**−1.73**	**−1**	**0**	**+1**	**+1.73**
**X1: Carbon source (%)**	0.63	1	1.5	2	2.37
**X_2_: Nitrogen source (%)**	0.63	1	1.5	2	2.37
**X3: Sea salt (%)**	0.13	0.5	1	1.5	1.87

CCD: Central composite design.

### Eukaryotic cell culture

All tested cell lines in the present work were cultured in Dulbecco’s Modified Eagles Medium (DMEM) supplemented with 2 *mM* L-glutamine, 100 units *ml*^−1^ penicillin, 100 *μg ml*^−1^ streptomycin and 10 % fetal bovine serum (GIBCO, USA) and maintained in a humidified atmosphere containing 5% CO_2_ at 37*°C*. The cells were serially passaged twice a week.

### Toxicity assessment

A cell viability assay was performed using the MTT method 23. Briefly, exponentially growing cells (10^4^/well) were seeded into 96 well flat bottom plates and incubated for 24 *hr* at 37°*C* in the presence of 5% CO_2_. Then, the cells were exposed to the C-137-R extract, in different ranges from 50–100 *μg/ml* concentrations in a period of 48 *hr*. DMSO (1%) and Prodigiosin (3 *μM*, more than its IC50 value) were used as negative and positive control, respectively. Afterwards, the MTT (3-(4,5-dimethylthiazol-2-yl)-2,5-diphenyltetrazolium bromide) solution was added to achieve a final concentration of 0.45 *mg ml*^−1^ followed by a further incubation for 4 *hr* at 37*°C*. Finally, equal volume of solubilization solution [40% (V/V)], dimethylformamide (DMF) in 2% (V/V) glacial acetic acid and 16% Sodium Dodecyl Sulfate (SDS) with pH ∼4.7 was added to each well to dissolve formazan crystals and the absorbance was recorded at 570 *nm* and 630 *nm* (Turbidity assessment) using the SPECTROstar^® Nano^ microplate reader (BMG LABTECH, Germany). Cell viability percentage was calculated by the following equation:

$$\begin{array}{c} \text{Cell}\;\text{viability}\;\%\;=\left(\frac{{\text{A}}_{\text{T}(\text{sample})}}{{\text{A}}_{\text{T}(\text{control})}}\right)\times 100\\ {\text{A}}_{\text{T}}={\text{A}}_{570}-{\text{A}}_{630}\\ \text{Cell}\;\text{death}\;\%\;=100-\text{cell}\;\text{viability}\;\% \end{array}$$

### Statistical analysis

Design Expert 8.0.7 was used for experimental design and MTT results analysis and graphing was performed by Graph Pad PRISM 6.0 software. The IC50 for the C-137-R extract was calculated using nonlinear regression analysis. All the experiments were performed in triplicate.

## Results

**Figure 1.**
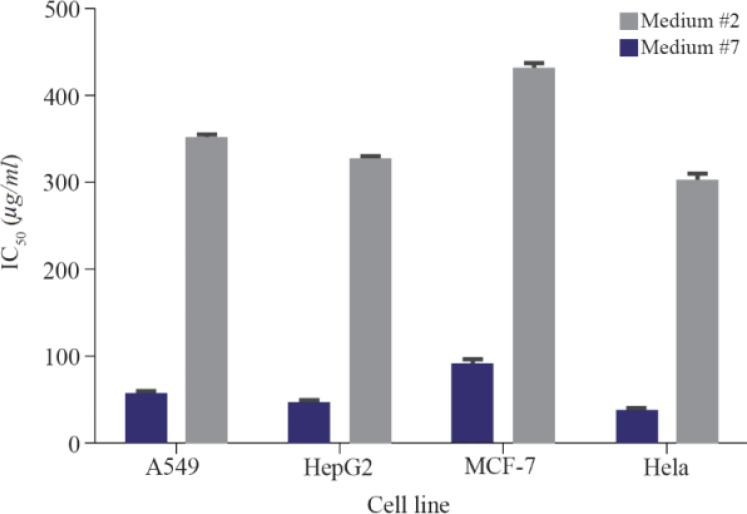
IC_50_ values of the C-137-R cytotoxic extract produced by *Bacillus velezensis* strain RP137 in carbon/nitrogen source selection experiments in media number 2 and 7 (The best and the worst response, respectively) against different cancerous cell lines.

To investigate the effect of low-cost medium components on the C-137-R production by *B. velezensis* strain RP137, a matrix of nine experiments was designed. Cytotoxicity of the C-137-R was evaluated on A549 and HepG2 cell lines. As shown in table 1, the best bacterial growth (Biomass) and organic extract production were observed when molasses and peptone were used (No #9), but the IC_50_ value of the obtained organic extract in this condition in A549 and HepG2 cells were 171.6 and 167.2 *μg/ml*, respectively (low cytotoxic effect). However, the lowest IC_50_ (54.4 and 45.1 *μg/ml* on A549 and HepG2 cells, respectively) belongs to the medium No #2 (Rice starch and KNO_3_ as carbon and nitrogen sources, respectively). Consequently, medium No #2 was selected as the best medium for the production of cytotoxic metabolite, C-137-R. As shown in figure 1, effect of the C-137-R on four cancerous cell lines (A549, HepG2, MCF-7, and Hela) was almost similar in the best (No #2) and the worst (No #7) media. So, further optimization procedures were performed only on HepG2 and A549 cell lines and analyzed by statistical approaches. In the following, the importance of the six parameters, namely, rice starch concentration, KNO_3_ concentration, sea salt concentration, inoculation size, pH and incubation time for the cytotoxic metabolite production was investigated by fractional factorial design. Tables 2 and 3 show the effects of these factors on the response and their significant levels. Based on the statistical analysis (Table 3), effects of rice starch and KNO_3_ concentrations were positive (Run No #4) and the sea salt concentration had negative effect (Run No #8), while all had confidence levels above 90%. So, they were identified as main factors affecting the C-137-R production. Incubation time, inoculation size and pH had less significant effects and were fixed in further studies. Moreover, R^2^ was found to be 0.999, which means that the model could explain 99.9% of the total variations in the system.

**Figure 2.**
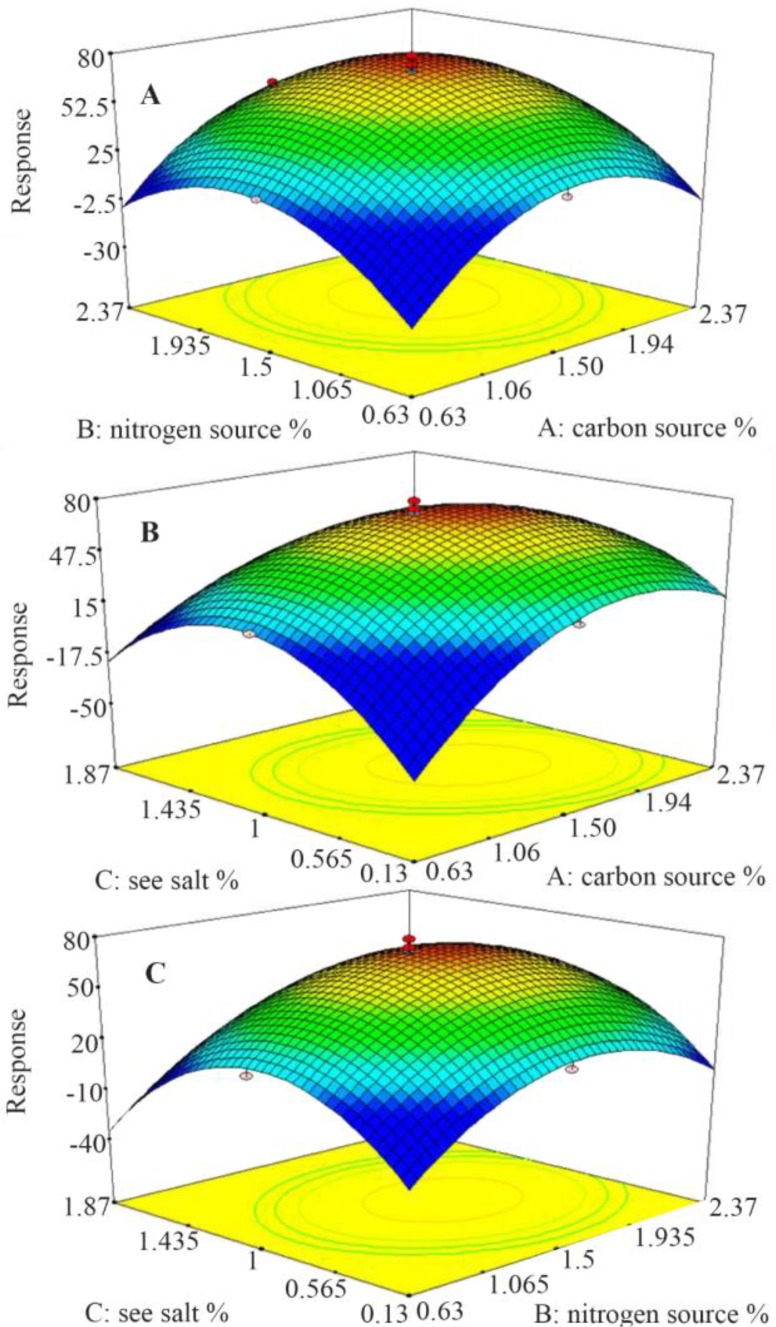
3D graphs of CCD experiments and the interaction between the variables.

The C-137-R synthesis by *B. velezensis* strain RP 137 was further optimized through central composite design (CCD) of response surface methodology. Twenty experiments were conducted at five different levels (Tables 4 and 5) with a 2^3^ factorial design and six axial points (α=1.73) and six replicates at the central point. The data were analyzed and a second-order polynomial equation was obtained to express the responses as a function of the independent variables as follows:

$$\text{Response}=71.45+7.84X_{1}+6.66X_{2}-4.14X_{3}-6.06X_{1}X_{3}-13.17X_{1}^{2}-11.92X_{2}^{2}-16.95X_{3}^{2}$$

where X_1_, X_2_ and X_3_ represent rice starch, KNO_3_ and sea salt concentrations in terms of coded factors, resectively. The positive coefficients of X1 and X2 and the negative coefficient of X3 in the response equation confirmed the positive effects of rice starch and KNO3 concentrations and the negative effect of sea salt concentraion. Analysis of varience (ANOVA) is presented in table 6. The model term is strongly significant at 99% confidence level (p<0.0001) with a R^2^ value of 0.927. The F value of 20.32 implies that there is only a 0.01% chance that this model could occur due to noise. In contrast to the negetive effect of sea salt concentration, the carbon and nitrogen sources had a significant positive effect on the C-137-R production (Run 9, 10, 11 and 12 in table 5). Moreover, the overal interaction effect of carbon and nitrogen sources on the response is positive (Run 4), indicating that enhancing the concentrations of rice starch and KNO_3_ leads to increase of the cytotoxic metabolite (C-137-R) production by the strain RP137. Figure 2 also reveals the positive interaction of the carbon and nitrogen sources (Figure 2A) and the negative effect of higher concentration of sea salt (Figure 2B and C). As shown in table 5, the concentrations chosen for the central points (1.5% rice strach and potassium nitrate and 1% sea salt) have the best effect on final responses with the mean response value of 71.45% (Run 15–20).

**Table 5.** CCD experimental design and the responses. The data are the average of at least three experiments ± standard deviation

**Run**	**X_1_**	**X_2_**	**X_3_**	**Extract D.W. (*mg/l*)**	**Cell Death HepG2 (%)**	**Cell Death A549 (%)**	**Response A549 (%)**
**1**	−1	−1	−1	66.7	11.5±4.9	15.6±3.5	5.8
**2**	+1	−1	−1	93.4	96.5±4.8	98.3±1.7	51.3
**3**	−1	+1	−1	70.0	99.3±4.8	97.4±2.5	38.2
**4**	+1	+1	−1	93.4	95.3±5.6	98.5±1.3	48.8
**5**	−1	−1	+1	106.7	46.9±2.0	40.2±4.5	23.9
**6**	+1	−1	+1	158.9	28.4±4.4	23.1±4.8	20.5
**7**	−1	+1	+1	76.7	56.5±3.9	50.8±3.4	21.8
**8**	+1	+1	+1	178.8	36.1±0.2	32.8±1.5	32.8
**9**	−1.73	0	0	55.6	32.6±3.9	27.5±2.0	17.3
**10**	+1.73	0	0	122.2	77.26±0.9	64.2±1.1	43.9
**11**	0	−1.73	0	91.2	38.5±2.0	37.4±4.8	19.0
**12**	0	+1.73	0	126.7	80.03±2.8	70.2±0.2	49.7
**13**	0	0	−1.73	83.3	51.6±5.3	49.9±14.2	23.0
**14**	0	0	+1.73	171.2	19.02±0.6	16.2±7.3	15.5
**15**	0	0	0	126.7	97.7±3.8	99.2±0.1	70.3
**16**	0	0	0	130.0	98.9±1.7	95.4±4.2	69.4
**17**	0	0	0	126.7	95.0±1.4	97.3±2.6	68.9
**18**	0	0	0	124.5	99.9±5.4	97.4±3.3	67.8
**19**	0	0	0	135.6	97.3±0.9	97.0±2.0	73.6
**20**	0	0	0	144.4	93.8±4.5	97.5±3.0	78.7

CCD: Central composite design, D.W.: Dry weight.

**Table 6.** Analysis of variance in CCD experiments

**Source**	**Sum of square**	**df**	**Mean square**	**F value**	**p-value**	
**Model**	9693.45	7	1384.79	27.77	<0.0001	Significant
**X_1_-Carbon**	860.72	1	860.72	17.26	0.0013	Significant
**X_2_-Nitrogen**	621.43	1	621.43	12.46	0.0041	Significant
**X_3_- see salt**	241.04	1	241.03	4.83	0.0482	Significant
**X_1_X_3_**	294.03	1	294.03	5.88	0.0318	Significant
**X_1_^2^**	2733.73	1	2733.73	54.83	<0.0001	Significant
**X_2_^2^**	2239.59	1	2239.59	44.92	<0.0001	Significant
**X_3_^2^**	4529.25	1	4529.23	90.84	<0.0001	Significant
**Residual**	598.29	12	49.85			
**Lack of Fit**	515.75	7	73.67	4.46	0.0594	Not significant
**Pure Error**	82.54	5	16.50			
**Core total**		19				

CCD: Central composite design.

**Figure 3.**
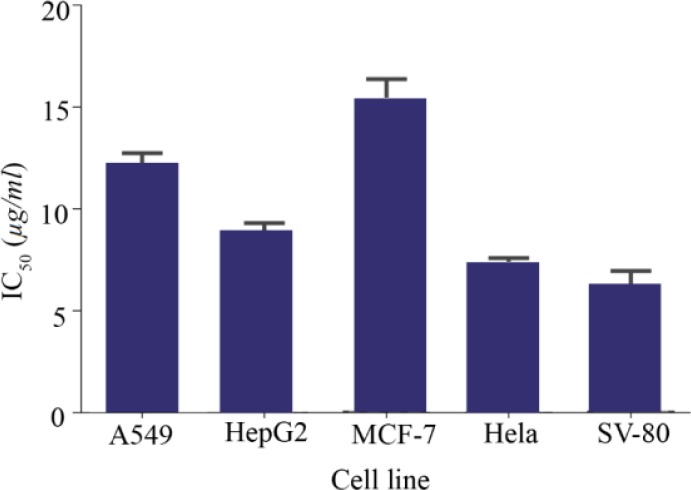
IC50 values of the C-137-R extract after maximum optimization in four cancerous cell lines and a normal cell line (SV-80).

Finally, cytotoxicity of the C-137-R extarct after optimization was evaluated on different cancerouce cell lines (A549, HepG2, MCF-7 and Hela) and human normal fibroblast cells (SV-80). As shown in figure 3, the highest IC50 value was obtained when the C-137-R was tested on MCF-7 cells (15.3±1.1 *μg/ml*) and the lowest was on Hela and SV-80 cells (7.3±0.4 and 6.2±1.0 *μg/ml*, respectively). Normal cells are sometimes more sensitive to toxic compounds, while cancerous cell lines such as MCF-7 show a potent drug resistance mechanism 24. The IC_50_ value determined on A549 cell line was 12.3 *μg/ml* which showed 28-fold optimization in C-137-R production by *B. velezensis* strain RP137.

## Discussion

Nutritional requirements play a crucial role during metabolite biosynthesis in microorganisms 25 and the availability of precursors inside cells might regulate secondary metabolite production. Among various nutrients, carbon and nitrogen sources are considered as important factors of metabolism, and many reports are available in literature which attempted to optimize the secondary metabolite production through alteration of medium components 18,26. In nature, nutrients are usually present in a wide range of complexity and abundance, and microorganisms must have tools to sense and use them to adjust their growth and metabolite production. Furthermore, when a mixture of carbon energy sources is presented in medium, microorganisms preferentially use those they can metabolize most effectively and easily and there are differences between the best carbon sources for growth or for secondary metabolism. For example, glucose is an excellent source for growth, but it is well established that it might suppress the secondary metabolite production 27–29. Other carbohydrates like maltose, glycerol, man-nose, xylose and sucrose have also been reported to effect the production of secondary metabolites negatively while complex carbon sources such as starch, wheat flour and molasses are good candidates for enhancing the metabolism 29–32. The same trend has been reported for nitrogen sources and several studies have shown that nitrogen assimilation is important for the regulation of secondary metabolite production. Effect of nitrogen sources on secondary metabolism is dependent on several factors including for instance, the metabolic pathway, the producing organism, the type and concentration of nitrogen sources, *etc*; nevertheless, the mechanisms involved are not clearly understood 33. Lowering the cost of bioactive compound production is of great demand in its biopharmaceutical applications. In many cases, the cost of production determines whether a pharmaceutical agent meets clinical use or not. In the present work, the effect of several complex carbon sources and different types of nitrogen sources on the production of C-137-R as a cytotoxic agent was investigated as indicated in table 1 and the best medium for the production was rice starch and KNO_3_. This result confirmed that a composition of complex carbon source and a simple nitrogen source can improve the C-137-R production by the strain RP137 along with lowering the cost of fermentation. Moreover, a similar trend of toxicity was observed on all four cancerous cell lines tested (Figure 1).

In comparison to other optimization methods, RSM requires fewer trials to investigate the variables and their interactions 20,34. The RSM approach has been adopted to improve the production of many pharmaceutical compounds in several microorganisms 35,36, revealing the impact of RSM strategies to maximize the yields of bioactive metabolites 37. Apart from medium composition, several other factors can influence the production of bioactive compounds including temperature, pH, inoculum size and the period of incubation. Temperature is a physiological factor that changes the fermentation process and synthesis of a variety of products 38,39. Furthermore, a low inoculation value may lead to insufficient biomass accumulation, leading to decrease in product formation. However, a coin has two sides, a high inoculation also can produce too much biomass and poor product formation 40. The effect of these variables in our fractional factorial experiments was investigated which revealed that the most significant factors are medium components and their concentrations were further optimized *via* RSM. Assessment of the final C-137-R extract in four cancerous cell lines and a normal cell line revealed its potent toxicity effect and excellent optimization of its production based on statistical approaches (Figure 3). The RSM designs conducted in the present investigation have been successfully applied in many recent biotechnological researches 32,41.

## Conclusion

In this study, as shown by fractional factorial and RSM results, a complex carbon source such as rice starch and a simple inorganic nitrogen source (KNO_3_) were significant factors with a positive effect in the C-137-R production by *B. velezensis* strain RP137. On the basis of data analysis, the concentration of medium components could be optimized appropriately and the cytotoxic metabolite production could be maximized 28 fold. The C-137-R showed promising potential as a cytotoxic extract and can be introduced as a good candidate for further studies on the purification and structural elucidation of its active metabolites in future.
